# Improving pediatric trauma care at a level 1 pediatric trauma center through the multi-year implementation of a Pediatric Trauma Boot Camp curriculum

**DOI:** 10.1186/s41077-025-00363-1

**Published:** 2025-06-17

**Authors:** Elizabeth M. Brigham, Erica I. Hodgman, Nicole A. Shilkofski, Justin M. Jeffers, Daniel An, Sean Tackett, Isam W. Nasr, Amanda B. Levin

**Affiliations:** 1 West Virginia University Children’s Hospital, Morgantown, WV USA; 2 Johns Hopkins University School of Medicine, Baltimore, MD USA; 3 Johns Hopkins Bayview Medical Center, Baltimore, MD USA

**Keywords:** Pediatric trauma, Trauma resuscitation, Simulation, Video review

## Abstract

**Background:**

Traumatic injuries are a significant contributor to pediatric morbidity and mortality, and trauma care necessitates that providers from different specialties and backgrounds be prepared to work together in high acuity settings to provide optimal care. Simulation-based trauma education consistently demonstrates improved knowledge, skill acquisition, teamwork, and task performance among providers, but relatively few studies assess provider performance during real resuscitations. The objective of this study is to develop an interdisciplinary pediatric trauma curriculum to improve trauma bay teamwork and adherence to ATLS ideals in the clinical environment.

**Methods:**

We developed a simulation-based pediatric trauma curriculum (Pediatric Trauma Boot Camp) incorporating learners from multiple departments and divisions all of whom care for pediatric trauma patients at our institution. To determine the impact of the curriculum on trauma team clinical performance, videos of trauma activations throughout the multi-year implementation period were reviewed and data abstracted. Teamwork was assessed using the Trauma NOTECHS scale and ATLS compliance by the presence or omission of eight items of the primary and secondary survey. Eighty-six total trainees participated during 2 years of curriculum implementation with faculty from General Pediatric Surgery, Pediatric Emergency Medicine, and Pediatric Critical Care serving as facilitators.

**Results:**

Out of a maximum of 25, the mean total Trauma NOTECHS score for the pre-pilot videos (*n* = 29) was 14.0. Post-pilot (*n* = 26), the mean total score improved to 16.8 (*p* = 0.001). Mean secondary survey completion improved from 4.1/8 pre-pilot to 5.4/8 post-pilot (*p* = 0.039). No significant difference was observed in primary survey completion between the first two cohorts. Following the second year of curriculum implementation, primary survey completion improved to 6.1/8 in the third cohort (*n* = 27) from 5.5/8 (*p* = 0.079). Continued improvement in total Trauma NOTECHS scores was observed (mean = 17.7), and improvements demonstrated in secondary survey completion were preserved.

**Conclusion:**

An interdisciplinary simulation-based pediatric trauma curriculum incorporating learners across specialties has the ability to positively impact provider behavior and direct patient care at a level 1 pediatric trauma center as evidenced by improved teamwork scores and secondary survey completion on video review of live trauma activations.

## Background

Traumatic injuries are one of the leading causes of pediatric morbidity and mortality in the USA [1]. The estimated total cost of inpatient hospitalizations for pediatric traumatic injuries between 2001 and 2011 was $21.69 billion [2]. It has been demonstrated, however, that management errors in pediatric trauma resuscitations are common and often involve basic resuscitation principles [3].

Pediatric trauma care is also inherently complex, involving multiple specialties working together to provide optimal care. One study demonstrated as many as 53 roles, 4 locations, and 69 pathways of pediatric care at a single institution [4]. Despite the collaboration required, and the ad hoc nature of the majority of trauma teams, training and education frequently occurs within individual departments.

In addition, injured children receive care in a variety of settings, including dedicated pediatric facilities, centers that treat both adults and children, and rarely, centers that have limited pediatric expertise. The Acute Trauma Life Support® (ATLS) course provides nationally recognized guidance for trauma care, but only a minority of its content focuses on the care of pediatric patients. No widely available comparable course exists which is specific to pediatrics, leaving a potential gap in education for trauma providers who may care for pediatric patients in a variety of settings.

Simulation is a frequently used tool to train physicians in trauma resuscitation. In addition to demonstrating improved knowledge, procedural skills, and non-technical skills, pediatric trauma centers that utilize a “high volume” of simulation training have been shown to have lower risk adjusted odds of mortality [5]. Qualitative data from the perspective of clinician participants has demonstrated the perceived utility of in situ pediatric trauma simulations to improve trauma team function [6]. Furthermore, multiple studies have demonstrated that simulation-based education improves the performance of pediatric trauma teams during simulated pediatric trauma resuscitations [7, 8]. Despite the consistent demonstration of improved knowledge, acquisition of non-technical skills, teamwork, and task performance following simulation-based trauma education in both adult and pediatric literature, relatively few studies assess provider performance and behavior beyond the simulated environment [9].

The subject of our research was a simulation-based trauma curriculum involving trainees who routinely participate in pediatric trauma activations at our institution. Facilitators included faculty from the divisions of Pediatric Emergency Medicine, General Pediatric Surgery, and Pediatric Critical Care. We hypothesized that this intervention would result in improved clinical performance as measured by teamwork assessment and primary and secondary survey completion at our level 1 pediatric trauma center.

## Methods

The setting for this study was a 209-bed pediatric hospital in a large metropolitan city which serves as the state’s level 1 Pediatric Trauma and Burn Center. Prior to this study, similar to other trauma programs nationally, our Pediatric Trauma Program facilitated and recorded both monthly in situ pediatric level 1 trauma simulations and actual pediatric trauma activations. The videos were collected for educational and performance improvement purposes and were reviewed in a general fashion by the trauma team without any formal data collection. Signage was posted in the trauma bay informing providers of the video-recording process and the use of videos for quality improvement purposes. This study was approved by our institution’s IRB as a quality improvement study (IRB00259232).

**Fig. 1:**
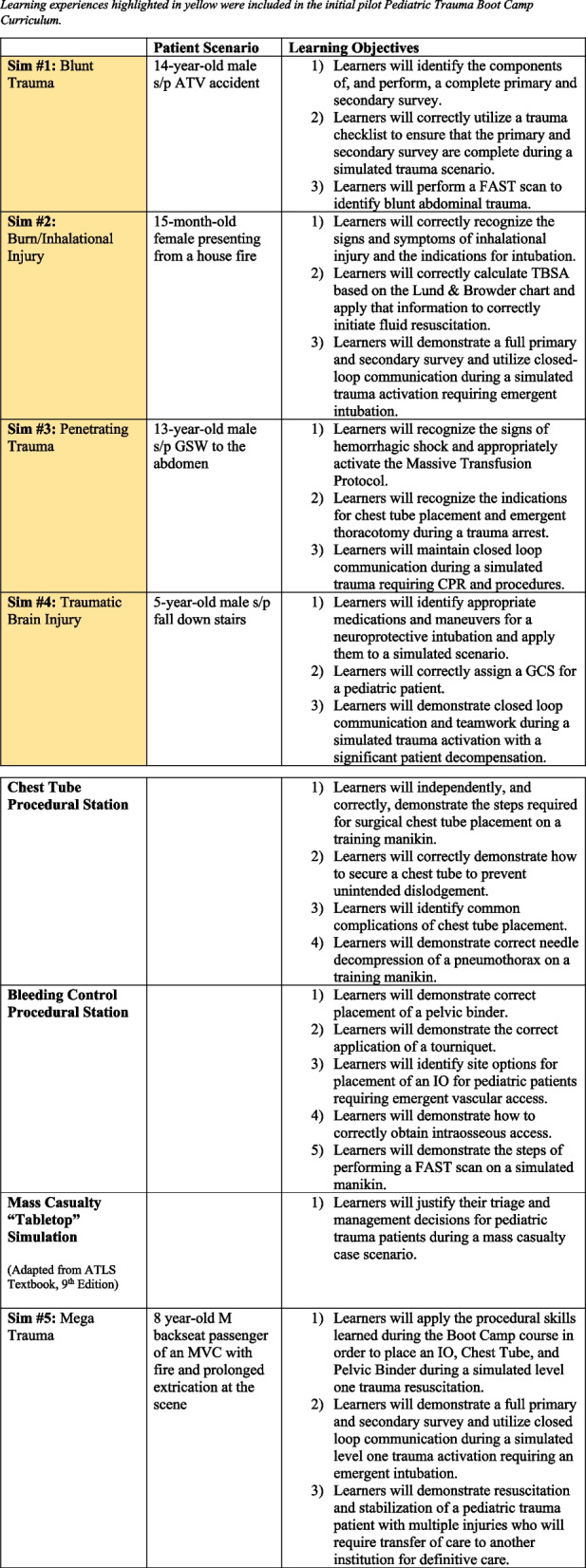
Summary of Pediatric Trauma Boot Camp curriculum. Learning experiences highlighted in yellow were included in the initial pilot Pediatric Trauma Boot Camp curriculum. Abbreviation key: ATV, all-terrain vehicle; FAST, Focused Assessment with Sonography in Trauma; TBSA, total body surface area; GSW, gunshot wound; CPR, cardiopulmonary resuscitation; GCS, Glasgow Coma Scale; IO, intraosseous (vascular access); MVC, motor vehicle collision

The intervention was an interdisciplinary pediatric trauma curriculum, known as the Pediatric Trauma Boot Camp. The half-day (4 h) pilot curriculum consisted of four commonly occurring pediatric trauma resuscitation scenarios written by trauma experts at our institution (Fig. 1). Participants included 19 fellows from the divisions of Pediatric Critical Care and Pediatric Emergency Medicine. Facilitators included 12 faculty from General Pediatric Surgery, Pediatric Critical Care, and Pediatric Emergency Medicine. Each scenario was facilitated by two faculty, each from a different division. Facilitators participated in 30-min virtual preparation sessions to review the learning objectives and scenarios prior to the course. Two identical sessions of the course were held (one in December 2020; one in January 2021) to capture all participants.

During the subsequent academic year (2021–2022), the Pediatric Trauma Boot Camp curriculum was expanded both in content and in the number of participants, including a wider variety of both trainees and faculty facilitators. In addition to the four simulated trauma resuscitation scenarios included in the pilot, a fifth “megatrauma” scenario was added, along with two procedural stations and a mass casualty “tabletop” simulation (Fig. 1). A pre-learning exercise consisting of watching and comparing videos of well-performed and poorly performed trauma activations was also added. A total of 67 additional learners and 23 facilitators participated in the full-day Boot Camp course (8 h), including residents from Emergency Medicine, General Surgery, and Pediatrics in addition to fellows in Pediatric Emergency Medicine and Pediatric Critical Care. Three identical sessions were held to capture all participants. Facilitators were again comprised of faculty from the divisions of General Pediatric Surgery, Pediatric Emergency Medicine, and Pediatric Critical Care.

**Fig. 2:**
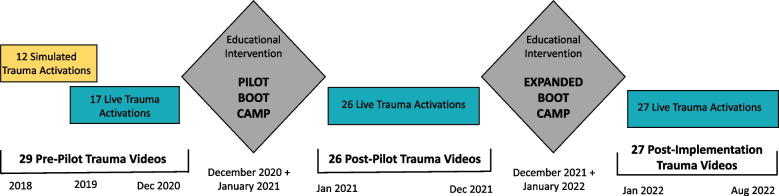
Study timeline

To determine the impact of Pediatric Trauma Boot Camp on trauma team clinical performance, videos of trauma activations throughout the implementation period were reviewed and data abstracted. All videos were of level 1 trauma activations as determined by institutional criteria and included a combination of blunt and penetrating traumatic injuries. Trauma activations with lower-level determinations were excluded. Twenty-nine videos were reviewed prior to the pilot intervention. Of this cohort, 12 videos were in situ simulations that took place during 2018–2020; the remaining 17 pre-pilot trauma videos were videos of live traumas, all of which took place within the 7 months prior to the implementation of the pilot curriculum in December 2020 (Fig. 2). In the post-pilot group, no simulated traumas were reviewed due to suspension of in situ simulation during the COVID-19 pandemic; post-pilot live trauma videos took place within the 11 months following the completion of the initial curriculum in January 2021. Following the expanded curriculum in December 2021/January 2022, an additional 27 post-implementation trauma videos were reviewed. Again, no simulated in situ traumas were included in this cohort due to COVID-19-related restrictions. We assessed teamwork using the Trauma NOTECHS scale and ATLS compliance by the presence or omission of eight items of the primary and secondary survey [10, 11]. Each video was scored by the same reviewer for consistency.

The NOTECHS scale is one of the most commonly studied leadership assessment tools utilized to evaluate healthcare action teams and one of the only scales specifically adapted for trauma [12]. A scoping review of tools utilized specifically for the assessment of trauma team performance identified Trauma NOTECHS as one of a minority of scales that encompasses all the critical themes recommended by expert opinion [13]. Excellent intra-class correlation has been demonstrated for total Trauma NOTECHS scores obtained via video review [14]. The Trauma NOTECHS scale consists of five domains (leadership, cooperation and resource management, communication and interaction, assessment and decision-making, and situational awareness/coping with stress) each assessed along a 5-point Likert type scale and combined for a total score [10]. The eight primary survey items and eight secondary survey items utilized to measure ATLS compliance have also been previously utilized to assess trauma team performance via video review [11]. In order to be counted as complete, each survey item had to be verbalized out loud by a member of the trauma team. During our own secondary review of a subset of trauma videos, we demonstrated a percent agreement of 73% and 86% respectively for the primary and secondary survey scores between two trained reviewers, an acceptable threshold for inter-rater agreement [15, 16]. Agreement was defined as an identical score ± 1 to account for the variation in video quality.

All data was summarized with descriptive statistics. Total Trauma NOTECHS scores were calculated by adding together the item responses for each of the five domains. We compared pre- and post-intervention means for each of the five individual Trauma NOTECHS domains and overall scores using* t*-tests before and after each of the two educational interventions. We also compared mean pre- and post-intervention total scores for primary and secondary survey completion (each out of a total of 8) using *t*-tests. Fisher’s exact tests were used to compare the percent completion of each primary and secondary survey item between the cohorts. Statistical analyses were performed using Stata (StataCorp. 2013. State Statistical Software: Release 13. College Station, TX: StatCorp LP).

## Results

For the 29 videos reviewed prior to the pilot Boot Camp intervention, the mean total Trauma NOTECHS score for the pre-intervention videos was 14.0 out of 25 (SD = 3.2, range = 5–21). Within the five Trauma NOTECHS domains, the domain with the lowest mean score (2.1, SD = 1.0) was “assessment and decision-making.” The highest mean score was for “situational awareness/coping with stress” (3.2, SD = 0.9). There was no significant difference in the mean total Trauma NOTECHS scores or the mean scores for each individual domain when comparing the simulated versus live trauma activations in the pre-pilot cohort (Table 1).

**Table 1:** Trauma NOTECHS scores simulated vs. live trauma activations (pre-pilot)

NOTECHS domain	Mean score live trauma activations (*n* = 17)	Mean score simulated trauma activations (*n* = 12)	*p* value (*t*-test)
Leadership	3.1	2.4	0.129
Cooperation and resource management	2.8	3.0	0.576
Communication and interaction	2.9	3.1	0.476
Assessment and decision-making	2.4	1.8	0.168
Situational awareness/coping with stress	3.4	3.0	0.308
Total Trauma NOTECHS score	14.5	13.3	0.361

In the 29 trauma activations reviewed pre-intervention, all eight items of the primary survey were completed only twice. The average primary survey score was 5.5/8 (SD = 1.5, range = 2–8). The most commonly excluded item was obtaining a temperature (41% completed). The most consistently completed item was breath sound auscultation (93% completed). Only 62% of the teams assigned a Glasgow Coma Scale (GCS) score. The average secondary survey score was 4.1/8 (SD = 2.1, range = 1–7), and no team completed all eight items. The most commonly excluded item was an ear exam (25% completed). The most consistently completed secondary survey item was a spine exam (88% completed).

In the post-pilot cohort, the mean total NOTECHS score improved to 16.8 (SD = 2.3, range = 12–20, *p* = 0.001). There was a significant improvement in the mean score of each individual domain except for the “assessment and decision-making” domain which again had the lowest mean score (2.6/5) (Table 2). Mean secondary survey completion improved from 4.1/8 pre-intervention to 5.5/8 post-intervention (SD = 1.8, range = 2–8, *p* = 0.039). There was a significant improvement in completion of the chest exam (Table 3). No significant difference was observed in primary survey completion between the two cohorts. Similarly, there was no significant improvement in completion of any of the individual items within the primary survey.

**Table 2:** Trauma NOTECHS scores across cohorts

NOTECHS domain	Pre-pilot mean score (*n* = 29)	Post-pilot mean score (*n* = 26)	*p* value (*t*-test)	Post-implementation mean score (*n* = 27)	*p* value (*t*-test)*
Leadership	2.8	3.8	0.0001	3.9	0.633
Cooperation and resource management	2.9	3.4	0.018	3.6	0.144
Communication and interaction	3.0	3.4	0.018	3.5	0.763
Assessment and decision-making	2.1	2.6	0.113	2.9	0.352
Situational awareness/coping with stress	3.2	3.7	0.029	3.9	0.062
Total Trauma NOTECHS score	14.0	16.8	0.001	17.7	0.186

^*^Comparing the post-pilot cohort with the post-implementation cohort

**Table 3:** Pre-pilot vs. post-pilot primary and secondary survey completion

Primary survey item	Pre-pilot percent complete (*n* = 29)	Post-pilot percent complete (*n* = 26)	*p* value (Fisher’s exact test)
Airway	86%	88%	1.000
Breath sounds	93%	100%	0.492
Heart rate	52%	23%	0.051
Extremity pulses	90%	100%	0.238
Blood pressure	59%	42%	0.285
GCS	62%	69%	0.777
Pupil exam	69%	65%	1.000
Temperature	41%	65%	0.106
	**Pre-pilot mean (SD)**	**Post-pilot mean (SD)**	*p* **value (t-test)**
**Total primary survey score**	5.5 (1.5)	5.5 (1.1)	0.954
**Secondary survey item**	**Pre-pilot percent complete** ***(n = 24)***	**Post-pilot percent complete** ***(n= 24)***	*p ***value (Fisher’s exact test)**
Head/face exam	54%	58%	1.000
Ear exam	25%	54%	0.075
Eye exam	67%	71%	1.000
Neck/cervical spine exam	38%	46%	0.770
Chest exam	29%	71%	0.009
Abdominal exam	50%	71%	0.238
Extremity exam	63%	71%	0.760
Spine exam	88%	96%	0.609
	**Pre-pilot mean (SD)**	**Post-pilot mean (SD)**	***p*** **value (t-test)**
**Total secondary survey score**	4.1 (2.1)	5.4 (1.8)	0.039

When comparing the post-pilot cohort with the final post-implementation cohort of trauma videos that were reviewed following the second, expanded Pediatric Trauma Boot Camp curriculum in 2021/2022, the improvement in total Trauma NOTECHS scores as well as the scores for each domain was preserved and additional improvement was demonstrated. None of the additional improvements was statistically significant compared to the post-pilot cohort (Table 2), but all were statistically significant when compared to the initial baseline pre-pilot cohort. When looking at ATLS compliance following the implementation of the expanded curriculum, the mean primary survey completion score improved from 5.5/8 to 6.1/8 (*p* = 0.079). While this improvement, as well as those demonstrated among the individual items of the primary survey, was not statistically significant, when the post-implementation cohort was compared to the pre-pilot videos, there was a significant improvement in obtaining a temperature (78% completion compared to 41% pre-pilot; *p* = 0.007) and an improvement in GCS completion from 62% to 85%. The improvement demonstrated in secondary survey completion following the pilot intervention was preserved (Table 4).

**Table 4:** Post-implementation primary and secondary survey completion

Primary survey item	Post-implementation percent complete (*n* = 27)	*p* value (Fisher’s exact test)*
Airway	93%	0.669
Breath sounds	100%	N/A
Heart rate	26%	1.0
Extremity pulses	100%	N/A
Blood pressure	56%	0.414
GCS	85%	0.202
Pupil exam	74%	0.559
Temperature	78%	0.372
	**Post-implementation mean**	***p*** value (t-test)*
**Total primary survey score**	6.1 (1.2)	0.079
**Secondary survey item**	**Post-implementation percent complete** ***(n= 23)***	***p*** value (Fisher’s exact test)*
Head/face exam	74%	0.359
Ear exam	57%	1.0
Eye exam	83%	0.494
Neck/cervical spine exam	39%	0.770
Chest exam	52%	0.238
Abdominal exam	52%	0.238
Extremity exam	78%	0.740
Spine exam	91%	0.609
	**Post-implementation mean**	***p*** value (t-test)*
**Total secondary survey score**	5.3 (1.4)	0.807

^*^Comparing the post-pilot cohort with the post-implementation cohort

## Discussion

In this multi-year, pre- and post-intervention cohort study, we demonstrate the ability of an interdisciplinary simulation-based pediatric trauma curriculum to positively impact the clinical performance of pediatric trauma teams at a level 1 pediatric trauma center. Despite initially capturing only fellow-level trainees who make up a minority of our pediatric trauma team members, the implementation of our pilot curriculum was associated with a significant improvement in total Trauma NOTECHS scores and a significant improvement in secondary survey completion.

Fellow-level trainees were chosen as our initial pilot learners as they most commonly fulfill the role of trauma team leader and manage the patient’s airway (with direct supervision and assistance as necessary from faculty) during level 1 pediatric trauma activations at our institution. The improvement observed after this first pilot intervention suggests that targeting providers that fill team leadership roles during trauma resuscitation with educational interventions may be a worthwhile strategy for improving clinical performance. As we expanded our curriculum to include resident-level trainees who fulfill the role of completing the patient assessment, assisting with exposure, and placing orders during trauma activations, we demonstrated retention of our initial improvements and continued improvement in completing specific items of the primary and secondary survey.

Unique to our study is its use of multiple metrics of trauma team performance in an actual clinical environment (rather than a simulated environment). In doing so, we directly measure the behavior of our trauma teams, a level of impact above reaction and learning on Kirkpatrick’s model, second only to the measure of patient outcomes [17]. While there are a large number of studies demonstrating the benefits of simulation training and education within both adult and pediatric trauma, relatively few meet this standard [8]. Those that do frequently focus on the performance of trainees from a single department rather than the function of the entire multi-disciplinary team [18]. Our curriculum itself is unique in its focus on bringing together trainees and faculty facilitators from multiple divisions, all of whom are involved in the care of pediatric trauma patients both during the resuscitation phase and during their continued care in the hospital. While not quantifiable, anecdotal feedback suggests that camaraderie between learners and divisions has improved as a result of these efforts, especially during the COVID-19 pandemic where isolation in divisions was the norm.

The weaknesses demonstrated by our pediatric trauma teams prior to the implementation of this curriculum are similar to those noted in other studies and at other institutions. For example, the original study utilizing the same primary and secondary survey checklist also noted a weakness in GCS completion with only 72.6% of teams completing that essential item of the primary survey [11]. Given the demonstrated correlation between initial GCS score and clinically important traumatic brain injuries, omitting this portion of the primary survey could put trauma teams at risk of missing a significant injury, and is an important deficit to target through educational efforts [19]. Similar to our trauma teams, breath sound auscultation was the most commonly completed item of the primary survey. The similarities between our trauma teams and those at another institution suggest that this curriculum could be generalized with potential educational benefits in other pediatric trauma programs.

The Trauma NOTECHS scale has demonstrated correlation with other clinical quality measures for trauma teams such as time to key tasks of a resuscitation, but little baseline data is available to pinpoint what a “good” score is for pediatric trauma teams [10]. One study recorded an average NOTECHS score of 20.5 among adult teams, suggesting that there is room for improvement within our trauma teams, and that the observed improvement in this study is of benefit to our patients [20].

One limitation of our study is an inability to control for the composition of any given trauma team responding to a level 1 pediatric trauma activation. For privacy, we did not include the name or role of any specific individual in our data collection; therefore, we have no knowledge of which specific trauma activations were attended or led by a trainee that completed the Pediatric Trauma Boot Camp curriculum. The ad hoc nature of trauma team activations and the large number of potential team members, including trainees visiting from other institutions, made training all possible team members impractical. It is also possible that the faculty facilitators’ participation in teaching the Boot Camp curriculum positively impacted their own contributions to clinical care in the trauma bay in addition to those of participating trainees.

We were also limited in our ability to include nurses and respiratory therapists in our two Boot Camp courses due to the staffing shortages that accompanied the COVID-19 pandemic. As vital members of the trauma team, we would expect their participation to further benefit team performance, and we did not measure the impact of our intervention on team culture or obtain feedback from team members who did not complete the curriculum. The results of our study should not be considered an endorsement of excluding any portion of the trauma team from educational interventions. Literature surrounding other types of hospital resuscitation teams has demonstrated that the integration of different types of providers in education and as part of leadership is one of the core themes among top-performing hospitals [21]. We plan for future iterations of our curriculum to continue to include a greater number and variety of our trauma team members, and we encourage other institutions to aspire to the same.

Our pre- and post-pilot data also differs as we had a mixture of in situ simulated and actual trauma activations in our pre-pilot data cohort while our post-pilot and post-implementation data includes only actual trauma activations; this was due to our inability to continue in situ simulation during the COVID-19 pandemic. Of note, no significant difference was noted in the Trauma NOTECHS scores for teams participating in simulated versus live trauma activations during the initial baseline cohort. Additionally, improved intra-class correlation for the NOTECHS scale has been demonstrated with the use of three reviewers, which we did not replicate [14]. Because one of our institution’s privacy controls is that no live trauma videos are retained longer than 30 days following the trauma activation, we were unable to blind the reviewer to the date or cohort of our trauma videos. For the ATLS compliance portion of our data collection, we awarded credit only to teams that verbalized the item of the primary or secondary survey out loud. This provided consistency in our data collection and was consistent with the goal of closed loop communication for resuscitation teams, but may not have captured some of the implicit communication that high functioning teams may utilize.

Our future plans include the continued annual implementation of the Pediatric Trauma Boot Camp curriculum with the goal of continuing to capture an increasing number of trainees and expand to other disciplines involved in trauma care. We will continue to refine the curriculum in an iterative fashion in order to address gaps in knowledge and skills of learners and to address ongoing deficits in our post-implementation data such as primary survey adherence. We will continue to follow data on the clinical performance of our level 1 pediatric trauma teams as we capture a larger number of trauma providers.

## Conclusions

By moving beyond simulation-based evaluation to review the actual clinical performance of trauma teams at our institution, we demonstrate the ability of an interdisciplinary simulation-based pediatric trauma curriculum incorporating learners across specialties to positively impact provider behavior and direct patient care on a program-level at a level 1 pediatric trauma center.

## Data Availability

The datasets used and/or analyzed during the current study are available from the corresponding author on reasonable request.

## Abbreviations

ATLS: Acute Trauma Life Support®
COVID-19: Coronavirus disease 2019
GCS: Glasgow Coma Scale
IRB: Institutional Review Board
NOTECHS: Non-technical skills
